# Porcine Babesiosis Caused by *Babesia* sp. Suis in a Pot-Bellied Pig in South Africa

**DOI:** 10.3389/fvets.2020.620462

**Published:** 2021-01-06

**Authors:** Alida Avenant, Janice Y. Park, Ilse Vorster, Emily P. Mitchell, Angela M. Arenas-Gamboa

**Affiliations:** ^1^Department of Paraclinical Sciences, Faculty of Veterinary Science, University of Pretoria, Pretoria, South Africa; ^2^Department of Veterinary Pathobiology, Texas A&M University, College of Veterinary Medicine & Biomedical Sciences, College Station, TX, United States; ^3^Department of Veterinary Tropical Diseases, Faculty of Veterinary Sciences, University of Pretoria, Pretoria, South Africa

**Keywords:** anemia, babesiosis, *Babesia* sp. Suis, hemoglobinuria, icterus, pig, porcine

## Abstract

Babesiosis is a worldwide, tick-borne disease of economic importance in livestock caused by *Babesia* spp., which are hemoparasitic piroplasms that target the host erythrocytes. Cattle, dogs, small ruminants, and wild ruminants are the species most commonly affected, while in cats, horses, and pigs, it is less frequently reported. Although babesiosis has been observed worldwide, porcine babesiosis remains an uncommon disease with a very limited number of cases reported. Here, we describe a case in a 12-year old pot-bellied pig from South Africa that died after a history of anorexia and reluctance to rise for 2 days. A complete necropsy, blood smear cytology, reverse line blot (RLB) hybridization and 18S rRNA sequencing were performed. Numerous *Babesia* spp. hemoparasites and a moderate regenerative anemia were identified on blood smear, and a urine dipstick test yielded 4+ heme. Diffuse icterus and splenomegaly were observed upon gross examination. Histopathology revealed hemoglobin casts within renal tubules and collecting ducts, pulmonary edema, splenic congestion, and intrahepatic cholestasis. BLASTN homology of the 18SrRNA sequence revealed a 100% identity to the published sequence of *Babesia* sp. Suis isolated from pigs in Italy. This case of babesiosis in a pig highlights the clinical manifestations and gross and pathological findings of porcine babesiosis.

## Introduction

Babesiosis, caused by *Babesia* spp., is a tick-borne disease of economic importance in livestock. This disease is found worldwide but is most prevalent in tropical and subtropical regions, where climatic conditions are more favorable for ticks (1). *Babesia* spp. infect and multiply within red blood cells, and the continual replication and exit of the intraerythrocytic piroplasms results in erythrocyte lysis. A complex interaction of direct and indirect mechanisms of erythrocyte damage result in hemolytic anemia and clinical signs of icterus, hemoglobinuria, and nephrosis (2–5). Anorexia, anemia, lethargy, lameness, reluctance to stand, high fever, and abortion manifest secondary to reduced oxygen-carrying capacity and compromised tissue perfusion (3, 6). Case fatality rates can be significant in severe acute infection or chronic infection without treatment.

Babesiosis affects a wide range of domestic vertebrates including humans. Cattle (*B. bigemina, B. bovis*), dogs (*B. canis, B. gibsoni, B. rossi, B. vogeli*), and small ruminants (*B. motasi, B. ovis*) are the most commonly affected species. Disease in cats (*B. felis*), horses *(B. caballi)*, and pigs is uncommon (1, 3, 7, 8). Swine babesiosis is primarily caused by *B. trautmanni* or less frequently by *B. perroncitoi* (9). Porcine babesiosis is rare in southern Africa, but two outbreaks were historically reported in domestic pigs in Limpopo Province, South Africa and Zimbabwe (9, 9–11). The causative species, *B. trautmanni*, was identified by the size and morphology of the parasites in blood or splenic smears (9–11).

This report describes gross and histopathological changes in a 12-year old, pot-bellied sow diagnosed with babesiosis following a brief course of anorexia and reluctance to rise for 2 days. The animal was treated by the owners for suspected constipation, but the pig subsequently died and was submitted to the Department of Paraclinical Sciences at the University of Pretoria for post-mortem examination. Babesiosis was diagnosed based on peripheral blood cytology, and the species was identified by reverse line blot (RLB) hybridization and sequence analysis of partial 18S rRNA isolated from the spleen.

The genetic diversity of *Babesia* species in pigs is largely unexplored, but recent molecular studies have identified a new subspecies, *Babesia* sp. Suis, in infected domestic pigs and ticks (6, 12) This report describes the first recognition of porcine babesiosis due to *B*. sp. Suis in a pet pot-bellied pig from Gauteng province, South Africa.

## Materials and Methods

A full necropsy was performed, and samples of the lungs, liver, kidneys, spleen, and heart were collected in 10% buffered formalin for histological examination. Post-mortem blood smears were stained with Kyro-Quick [Kyron Laboratories (Pty) Ltd] Romanowsky stain to evaluate cytology. Formalin-fixed samples were routinely processed and stained with hematoxylin and eosin.

Fresh tissue from the spleen was analyzed for the presence of *Theileria* and *Babesia* spp. using the RLB hybridization assay. Genomic DNA extracted using the Purelink™ Genomic DNA Mini Kit (Invitrogen by Life Technologies™, United States) was subjected to PCR using the *Theileria*/*Babesia* genus–specific primers RLB F2 [5′-GAC ACA GGG AGG TAG TGA CAA G-3′] and biotin labeled RLB R2 [5′-Biotin-CTA AGA ATT TCA CCT CTA ACA GT-3′] (13–15). Reactions were performed in 25 μl volume containing 12.5 μl of 2X Phusion Flash High-Fidelity PCR Master Mix (Thermo Scientific, LTC Tech, South Africa), 0.2 μM of each primer (forward and reverse), 9.5 μl nuclease-free water, and 2.5 μl of DNA template (between 50 and 100 ng DNA). *B. bigemina* DNA (Onderstepoort Biological Products, South Africa) was included as a positive control; the negative control was PCR master mix without template DNA. PCR amplification was performed under the conditions described in Supplementary Table 1, and the products were hybridized on the RLB membrane, as described by Gubbels et al. (13), to the genus- and species-specific oligonucleotide probes summarized in Supplementary Table 2.

RLB results were confirmed by cloning and sequencing a partial sequence (~750 bp) of the 18S rRNA gene using BT2-F (5′-GGA GTA TGG TCG CAA GTC TG−3′) and BT2-R (5′-ctt ctg cag gtt cac cta cg-3′) (16) primers. PCR reactions were performed as described above with the following conditions: initial denaturation of 10 s at 98°C, followed by 35 cycles of 1 s at 98°C, 5 s at 61°C, and 15 s at 72°C, and a final extension of 1 min at 72°C.

PCR amplicons were purified using the Purelink™ Quick Gel Extraction and PCR Purification Combo Kit (Invitrogen by Life Technologies™, United States). Using the CloneJET PCR Cloning Kit (Thermo Scientific, LTC Tech, South Africa), the purified amplicons were ligated into the pJET1.2/blunt cloning vector and transformed into competent *Escherichia coli* JM109 High Efficiency Competent cells (Promega, Madison, USA), according to the manufacturer's instructions. Plasmid isolation was done using the PureLink™ HiPure Plasmid Miniprep Kit (Invitrogen by Life Technologies™, United States), and 3 recombinant plasmids were sequenced on an ABI 3500XL Genetic Analyzer, using the vector primers pJET1.2_F and pJET1.2_R, at Inqaba Biotechnical Industries (Pty) Ltd (Pretoria, South Africa).

Sequences were assembled and edited using the Staden software suite (17). The sequences were compared to the GenBank database for homologous sequences using nucleotide blast BLASTN (18). Phylogenetic analyses were performed by aligning the partial 18S rRNA sequences obtained from this case with a set of 20 sequences (Supplementary Table 2), mainly representative of the Babesidae clade VI, as previously described (6, 12, 19). The Ungulibabesids, as previously defined by Criado-Fornelio et al. (16) fall within this clade of Babesiadae and is where *Babesia* sp. Suis is currently grouped. The tree was rooted using the 18S rRNA sequence of *Toxoplasma gondii* (X68523.1).

A multiple sequence alignment was performed with the above-mentioned sequences using the Multiple Alignment Fast Fourier Transform (MAFFT) (version 7) (20, 21). The alignments were manually truncated, using BioEdit (version 7. 2) (22), to the length of the shortest sequence. The evolutionary history was inferred by using the Maximum Likelihood method and General Time Reversible model (23). Phylogenetic trees were constructed by the neighbor-joining, maximum-likelihood and maximum-parsimony methods using MEGA X (version 10.1.8) (24). Initial tree(s) for the heuristic search were obtained automatically by applying Neighbor-Joining (NJ) and BioNJ algorithms to a matrix of pairwise distances estimated using the Maximum Composite Likelihood (MCL) approach, and then selecting the topology with the highest log likelihood value. A discrete Gamma distribution was used to model evolutionary rate differences among sites [five categories (+*G*, parameter = 0.1986)]. The rate variation model allowed for some sites to be evolutionarily invariable [(+ I), 37.13% sites]. The tree was drawn to scale, with branch lengths measured in the number of substitutions per site. The analysis involved 22 nucleotide sequences. Bootstrap analysis was carried out for each method using a 1000 iterations per tree (25). Evolutionary analysis was conducted in MEGA X (24).

## Results

**Figure 1:**
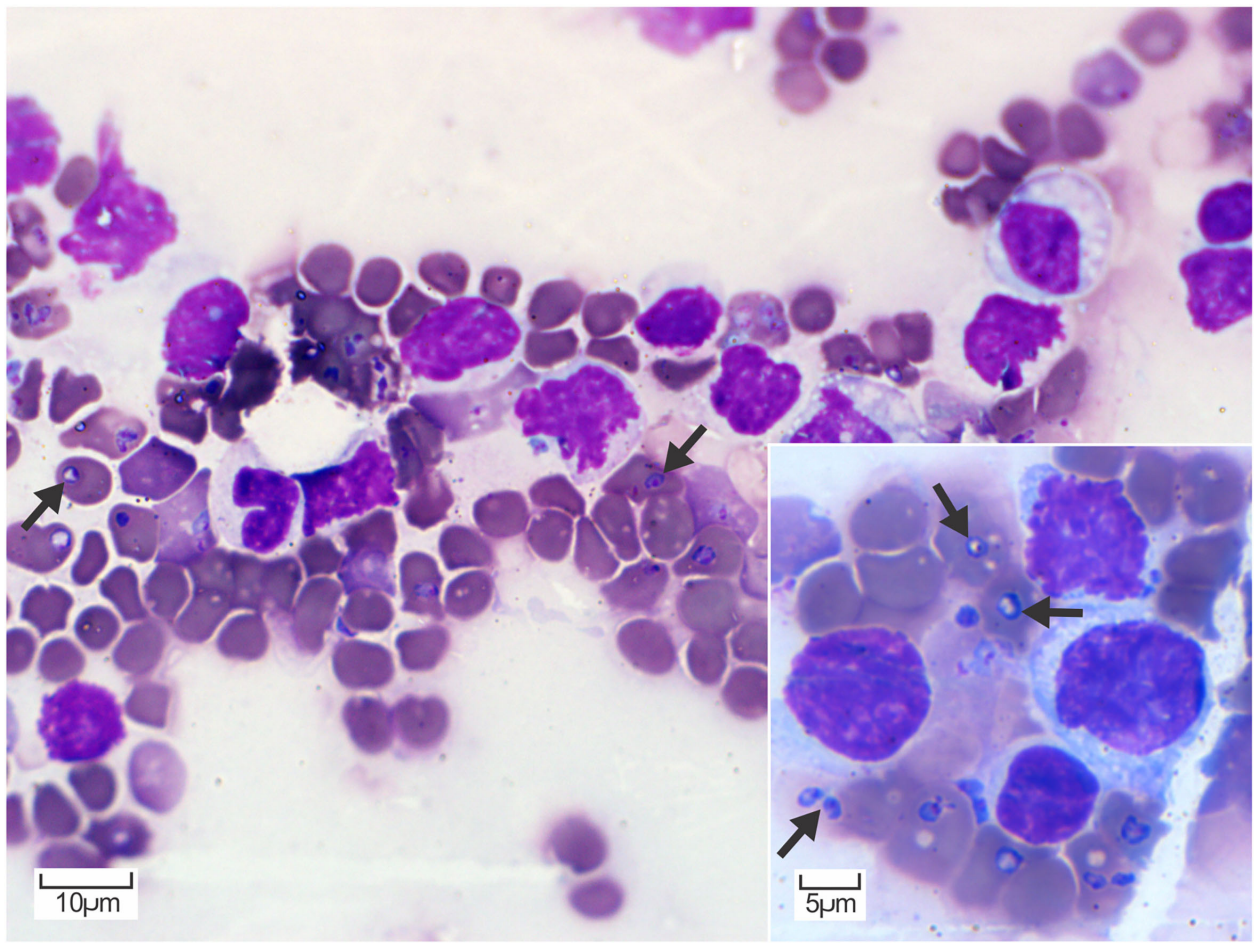
Peripheral blood smear cytology. Erythrocytes contain multiple, round to piriform, 2–3 μm, pale basophilic piroplasms with a small amphophilic nucleus (arrows). Diff-Quik. Inset: Higher magnification of erythrocytes containing piroplasms consistent with *Babesia* spp. occurring as individual or occasionally paired structures (arrows). Diff-Quik.

At the postmortem examination, the blood was thin and watery. Blood smear cytology revealed 1 to 4, round- to piriform, protozoan piroplasms (2–3 μm) with round to oval eccentric basophilic to amphophilic nuclei and abundant pale basophilic cytoplasm, consistent with *Babesia* spp. in numerous erythrocytes (Figure 1). Erythrocytes had mild anisocytosis and polychromasia due to reticulocytosis demonstrating mild to moderate regenerative anemia.

The mucous membranes, fat, and the intimal surface of the aorta were yellow. Approximately 100 ml and 20 ml of yellow-tinged fluid were present within the thoracic cavity and pericardial sac, respectively. The lungs were congested and edematous with rib impressions on the pleural surface. Petechial hemorrhages were scattered on the pleural surface of the lungs and in the subepicardial myocardium of the right atrium. The spleen was moderately enlarged and congested. The kidneys were mildly enlarged and soft. The urine was opaque, olive green, and contained dark red- to black particles. Urine analysis noted 4+ heme, 2+ protein, and 1+ glucose.

**Figure 2:**
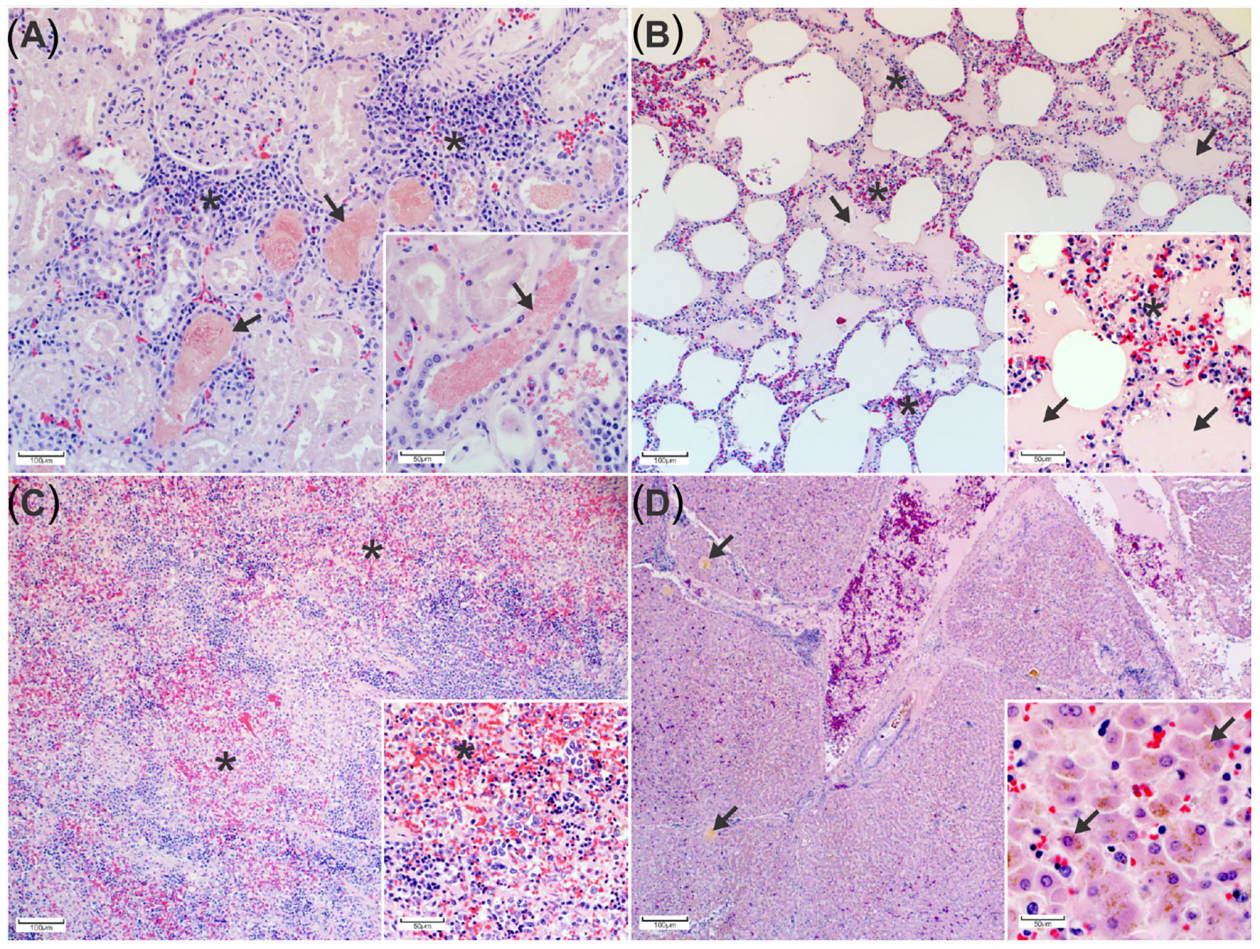
Histopathological changes associated with porcine babesiosis. **(A)** Kidney: Tubules and collecting ducts contain eosinophilic, finely granular hemoglobin casts. The interstitial space is expanded by extramedullary hematopoiesis (*). (H&E). Inset: Higher magnification of hemoglobin casts in tubules indicating hemoglobinuria (arrows) (H&E). **(B)** Lung: Alveolar spaces contain proteinaceous edema (arrows). The alveolar walls are expanded by mononuclear inflammatory cells (*) (H&E). Inset: alveolar edema and alveolar macrophage proliferation (arrows) (H&E). **(C)** Spleen with multiple areas of congestion (*) (H&E). Insert: splenic macrophage proliferation and extramedullary hematopoiesis (H&E). **(D)** Liver The hepatic canaliculi are expanded by bile (arrows) (H&E) Insert: bile pigment in hepatocytes (H&E).

Histopathology of the kidneys revealed eosinophilic, finely granular hemoglobin casts in numerous renal tubular lumens and collecting ducts accompanied by scattered foci of tubular degeneration and necrosis. Tubular epithelial cell regeneration was characterized by increased numbers of low cuboidal epithelial cells with large round to oval nuclei and pale basophilic cytoplasm (Figure 2A). In the lungs, the alveolar wall interstitium was expanded by lymphocytes and macrophages, and numerous alveoli contained proteinaceous edema fluid (Figure 2B). The spleen was congested and expanded by proliferation of macrophages (Figure 2C) and extramedullary hematopoiesis (EMH). Other areas of EMH included the renal cortical interstitium mainly at the corticomedullary junction and multifocally throughout the hepatic parenchyma and portal tracts. The liver displayed widespread intrahepatic canicular cholestasis (Figure 2D).

**Figure 3:**
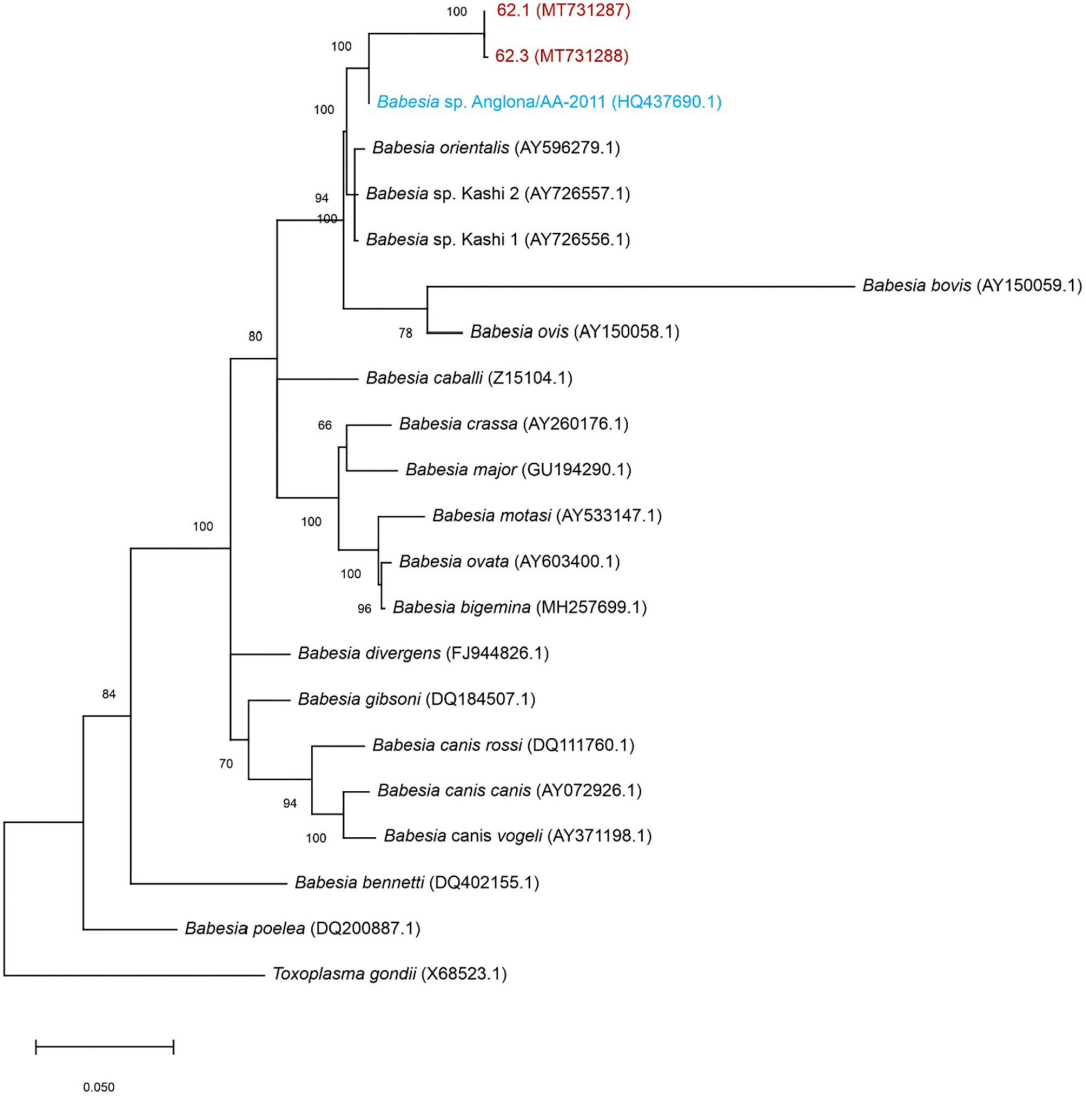
Maximum-likelihood tree based on 18S rRNA nucleotide sequences. The tree shows the phylogenetic relationship between the porcine isolates (MT731287 and MT731288-red), *B*. sp. Suis (HQ437690.1-blue) compared to other *Babesia* spp. *Toxoplasma gondii* was included as outgroup. The numbers at the internal nodes represent the percentage of 1,000 replicates (bootstrap) for which the same branching patterns were obtained. The percentage of trees in which the associated taxa clustered together is indicated next to the branches. The tree with the highest log likelihood (−3,933.11) is shown.

*Babesia* 18S rRNA in the spleen sample hybridized with the *Theileria*/*Babesia* genus-specific probe, *Babesia* catch-all 1 and 2 genus-specific probes, and *B. bigemina* species-specific probe (Supplementary Figure 1). The 18S rRNA sequence was then used for BLAST analysis and demonstrated a 100% sequence identity to *B*. sp. Anglona/AA-2011 (HQ437690.1). This species, referred to as *B*. sp. Suis, was originally isolated from a pig sample in Italy (6). Phylogenetically, the *Babesia* sequence obtained from the pig spleen is closely related to the Ungulibabesids clade along with *B*. sp. Suis, *B*. sp. Kashi 1 and 2, and *B. orientalis* (Figure 3).

## Discussion

Porcine babesiosis may be missed if peripheral blood smears are not performed or if babesiosis is not considered as a differential diagnosis in areas with historically low incidence of tick-borne disease. Gross findings such as icterus, anemia, and hemoglobinuria are non-specific, and differential diagnoses to consider in pigs included babesiosis, *Eperythrozoon suis*, and acute leptospirosis (9).

The pathogenesis of *Babesia* spp. starts with a tick taking a blood meal from a mammalian host (1, 7). The parasite attaches to and enters erythrocytes via endocytosis. At this stage, cytology can be used to identify parasites within erythrocytes on peripheral blood smears in live animals or tissues at necropsy. Blood smear cytology was a critical tool needed to diagnose *Babesia* spp. as the causative agent in this case, but sequencing was necessary to identify the species responsible (9). Sequencing of the partial 18S ribosomal RNA in this case was 100% similar to the sequence for *B*. sp. Suis (6).

The rapid and continual intraerythrocytic multiplication of *Babesia* spp. parasites results in intravascular and extravascular hemolytic anemia from erythrocyte lysis by emerging parasites (5, 26, 27). Intravascular hemolysis causes hemoglobinuria and acute tubular necrosis (28–31). Extravascular hemolysis also occurs leading to bilirubinemia and cholestasis of the liver, which manifests as icterus. Clinical symptoms of lethargy, lameness, and fever are secondary to reduced oxygen-carrying capacity from red blood cell loss (3, 6). EMH and regenerative anemia, characterized by reticulocytosis and polychromasia, are compensatory responses to erythrocyte destruction. Thrombocytopenia is a feature of babesiosis that results in petechial hemorrhage, but the mechanism of platelet damage is unknown (32, 33). Acute lung injury and pneumonitis is less common, but has been reported in dogs infected with *B. rossi* and in human cases (33–35).

The life cycle of *Babesia* spp. begins when a tick ingests *Babesia* gametes while feeding on infected animals. *Ixodes* spp. ticks are common vectors, but *Rhipicephalus* spp., *Haemaphysalis* spp., or *Dermacentor* spp. ticks may also vector *Babesia* spp. (7, 27). Once inside the tick, *Babesia* spp. undergo schizogony to form gametes within the tick's gut and then mature into infectious sporozoites within the salivary glands (7). Ticks pass sporozoites to new vertebrate hosts upon feeding, and the sporozoites differentiate into merozoites within host erythrocytes (3, 7). Transovarial transmission between ticks is also possible when *Babesia* spp. vermicules migrate to the female tick's ovaries (36).

The most common *Babesia* species identified in pigs is *B. trautmanni*, which is vectored by *Rhipicephalus* spp. ticks (6, 9, 36). The previous reports of babesiosis in southern Africa all occurred in pig production units in the 1940s−1950s, and the causative species reported based on morphologic features of the parasite in blood was *B. trautmanni* (10, 11). The natural hosts for *B. trautmanni* are thought to be the warthog (*Phacochoerus africanus*) and bushpig (*Potamochoerus larvatus*) (6, 36, 37). Intriguingly, based on piroplasm size and genetic homology, *B*. sp. Suis is a relative of *B. trautmanni*. Interestingly, icterus was not reported in any of the previous cases where *B. trautmanni* was identified as the causative species (9–11). Variation in disease severity and clinical manifestations may be due to pathogen factors or the response of the host to infection. Zobba et al. speculated that different strains identified in clinically ill and in asymptomatic pigs may be responsible for different degrees of pathogenicity (12).

The ability to successfully transmit babesiosis from one host to another depends on the specific invertebrate host and the vertebrate host's competency to maintain the agent in its infectious state (3). Climate change may exacerbate disease transmission events by redistributing the tick vectors' spatial and temporal ranges (38). External pressures may also change tick behavior and generate evolutionary pressure on the pathogen, which may affect host range or pathogenicity (39, 40). Climate variables such as temperature, humidity, and rainfall are known to influence adult tick activity (1, 38). Increasing environmental temperatures may expand tick geographical ranges and introduce the pathogen to naïve animal populations (40–42). As human populations grow and wildlife habitats are fragmented and reduced, increased contact between reservoir hosts (e.g., bushpigs and warthogs for *B. trautmanni*) and domestic pigs may increase transmission. Close proximity exposes animals to carriers, and the length of time of tick attachment is directly related to efficacy of transmitting *Babesia* spp. sporozoites (3).

## Conclusion

The gross findings, histological changes, and identification of *Babesia* sp. on blood smear were consistent with a case of babesiosis caused by a recently recognized species, *Babesia* sp. Suis. This case represents an unusual pathogen identified in an unusual host and emphasizes the increasing importance of public awareness in regions where porcine babesiosis has been historically rare, such as in southern Africa. Multiple environmental and evolutionary pressures on tick vectors and related causative agents are anticipated to cause widened spread of tickborne diseases. As a result, babesiosis should be considered a possible differential diagnosis for pigs that present with lethargy, anemia, icterus, and hemoglobinuria. Blood smears and molecular techniques will complement routine necropsy techniques in the diagnosis of sporadic and emerging protozoal diseases including babesiosis.

## Data Availability Statement

The datasets presented in this study can be found in online repositories. The names of the repository/repositories and accession number(s) can be found at: https://www.ncbi.nlm.nih.gov/genbank/, SUB7720577 62.1 MT731287, https://www.ncbi.nlm.nih.gov/genbank/, SUB7720577 62.3 MT731288.

## Author Contributions

AA: pathological examination, writing—original draft, and writing—review and editing. JP: writing—original draft and writing—review and editing. IV: molecular analysis, phylogenetic analysis, writing—original draft, and writing—review and editing. EM and AA-G: writing—original draft (supporting), writing—review and editing, and supervision. All authors contributed to the article and approved the submitted version.

## Conflict of Interest

The authors declare that the research was conducted in the absence of any commercial or financial relationships that could be construed as a potential conflict of interest.
