# Supplementary material for: Porcine Babesiosis Caused by Babesia sp. Suis in a Pot-Bellied Pig in South Africa
Source: Front Vet Sci. 2021 Jan 6;7:620462. doi: 10.3389/fvets.2020.620462 (PMC7815520; doi:10.3389/fvets.2020.620462)
Supplement: Supplementary file 1 [file Table_1.docx]

Table S1. Thermocycling conditions for Theileria/Babesia touchdown PCR

| Cycle | Time | Temperature | Purpose |
| --- | --- | --- | --- |
| 1 cycle | 3 min | 37°C |  |
| 1 cycle | 10 min | 94°C | Initial denaturation |
| 2 cycles | 20 sec 30 sec 30 sec | 94°C 67°C 72°C | Denaturation of double-stranded DNA template Annealing of primers  Extension of PCR products by *Taq* polymerase |
| 2 cycles | 20 sec 30 sec 30 sec | 94°C 65°C 72°C | Denaturation of double-stranded DNA template Annealing of primers  Extension of PCR products by *Taq* polymerase |
| 2 cycles | 20 sec 30 sec 30 sec | 94°C 63°C 72°C | Denaturation of double-stranded DNA template Annealing of primers  Extension of PCR products by *Taq* polymerase |
| 2 cycles | 20 sec 30 sec 30 sec | 94°C 61°C 72°C | Denaturation of double-stranded DNA template Annealing of primers  Extension of PCR products by *Taq* polymerase |
| 2 cycles | 20 sec 30 sec 30 sec | 94°C 59°C 72°C | Denaturation of double-stranded DNA template Annealing of primers  Extension of PCR products by *Taq* polymerase |
| 40 cycles | 20 sec 30 sec 30 sec | 94°C 57°C 72°C | Denaturation of double-stranded DNA template Annealing of primers  Extension of PCR products by *Taq* polymerase |
| 1 cycle | 7 min | 72°C | Final extension |
