# Supplementary material for: Porcine Babesiosis Caused by Babesia sp. Suis in a Pot-Bellied Pig in South Africa
Source: Front Vet Sci. 2021 Jan 6;7:620462. doi: 10.3389/fvets.2020.620462 (PMC7815520; doi:10.3389/fvets.2020.620462)
Supplement: Supplementary file 2 [file Table_2.docx]

Table S2. Genus- and species-specific RLB oligonucleotide probes used in this study

| Pathogen | Sequence (5'-3') | Reference |
| --- | --- | --- |
| *Ehrlichia/Anaplasma* genus-specific | GGG GGA AAG ATT TAT CGC TA | Bekker *et al.*, 2002 |
| *Anaplasma bovis* | GTA GCT TGC TAT GR^†^G AAC A | Bekker *et al.,* 2002 |
| *Anaplasma centrale* | TCG AAC GGA CCA TAC GC | Bekker *et al.,* 2002 |
| *Anaplasma marginale* | GAC CGT ATA CGC AGC TTG | Bekker *et al.*, 2002 |
| *Anaplasma phagocytophilum* | TTG CTA TAA AGA ATA ATT AGT GG | Bekker *et al.,* 2002 |
| *Anaplasma* sp. Omatjenne | CGG ATT TTT ATC ATA GCT TGC | Bekker *et al.,* 2002 |
| *Ehrlichia canis* | TCT GGC TAT AGG AAA TTG TTA | Schouls *et al.*, 1999 |
| *Ehrlichia chaffeensis* | ACC TTT TGG TTA TAA ATA ATT GTT | Schouls *et al.,* 1999 |
| *Ehrlichia ruminantium* | AGT ATC TGT TAG TGG CAG | Bekker *et al.,* 2002 |
| *Theileria/Babesia* genus-specific | TAA TGG TTA ATA GGA R^†^CR^†^GTT G | Gubbels *et al.*, 1999 |
| *Babesia* 1 genus-specific | ATT AGA GTG CTC AAA GCA GGC | Nijhof (unpublished) |
| *Babesia* 2 genus-specific | ACT AGA GTG TTT CAA ACA GGC | Nijhof (unpublished) |
| *Babesia bicornis* | TTG GTA AAT CGC CTT GGT C | Nijhof *et al.*, 2003 |
| *Babesia bigemina* | GTA GTT GTA TTT CAG CCT CG | Stoltsz *et al.*, 2020 |
| *Babesia bovis* | CAG GTT TCG CCT GTA TAA TTG AG | Gubbels *et al*., 1999 |
| *Babesia caballi* | GTG TTT ATC GCA GAC TTT TGT | Butler *et al.*, 2008 |
| *Babesia canis canis* | TGC GTT GAC GGT TTG AC | Matjila *et al.*, 2004 |
| *Babesia canis rossi* | CGG TTT GTT GCC TTT GTG | Matjila *et al*., 2004 |
| *Babesia canis vogeli* | AGC GTG TTC GAG TTT GCC | Matjila *et al*., 2004 |
| *Babesia divergens* | ACT R^†^AT GTC GAG ATT GCA C | Nijhof *et al.,* 2003 |
| *Babesia felis* | TTA TGC GTT TTC CGA CTG GC | Bosman, Venter and Penzhorn, 2007 |
| *Babesia gibsoni* | TAC TTG CCT TGT CTG GTT T | Yisaschar-Mekuzas *et al.*, 2013 |
| *Babesia leo* | TTA TGC TTT TCC GAC TGG C | Bosman, Venter and Penzhorn, 2007 |
| *Babesia major* | TCC GAC TTT GGT TGG TGT | Georges *et al.*, 2001 |
| *Babesia microti* | GR^a^C TTG GCA TCW^‡^TCT GGA | Nijhof *et al.,* 2003 |
| *Babesia occultans* | CCT CTT TTG GCC CAT CTC G | He *et al.,* 2012 |
| *Babesia* sp. (sable) | GCG TTG ACT TTG TGT CTT TAG C | Oosthuizen *et al.*, 2008 |
| *Theileria* genus-specific | ATT AGA GTG TTT CAA GCA GAC | Nijhof (unpublished) |
| *Theileria annae* | CCG AAC GTA ATT TTA TTG ATT G | Yisaschar-Mekuzas *et al.,* 2013 |
| *Theileria annulata* | CCT CTG GGG TCT GTG CA | Georges *et al.,* 2001 |
| *Theileria bicornis* | GCG TTG TGG CTT TTT TCT G | Nijhof *et al.,* 2003 |
| *Theileria buffeli* | GGC TTA TTT CGG W^‡^TT GAT TTT | Gubbels *et al.*, 2000 |
| *Theileria equi* | TTC GTT GAC TGC GYT TGG | Butler *et al*., 2008 |
| *Theileria lestoquardi* | CTT GTG TCC CTC CGG G | Schnittger *et al.*, 2004 |
| *Theileria mutans* | CTT GCG TCT CCG AAT GTT | Gubbels *et al.,* 1999 |
| *Theileria ovis* | TGC GCG CGG CCT TTG CGT T | Bekker *et al*., 2002 |
| *Theileria parva* | GGA CGG AGT TCG CTT TG | Nijhof *et al.,* 2003 |
| *Theileria separata* | GGT CGT GGT TTT CCT CGT | Schnittger *et al.,* 2004 |
| *Theileria* sp. (buffalo) | CAG ACG GAG TTT ACT TTG T | Oura *et al.*, 2004 |
| *Theileria* sp. (kudu) | CTG CAT TGT TTC TTT CCT TTG | Nijhof *et al.*, 2005 |
| *Theileria* sp. (sable) | GCT GCA TTG CCT TTT CTC C | Nijhof *et al*., 2005 |
| *Theileria taurotragi* | TCT TGG CAC GTG GCT TTT | Gubbels *et al*., 1999 |
| *Theileria velifera* | CCT ATT CTC CTT TAC GAG T | Gubbels *et al.,* 1999 |

^†^ The degenerate position R denotes either A or G

^‡^ The degenerate position W denotes either A or T

**REFERENCES**

Butler CM, Nijhof AM, Jongejan F, Van Der Kolk JH. *Anaplasma phagocytophilum* infection in horses in the Netherlands. *Vet Rec*. (2008) 162:216–8. doi: 10.1136/vr.162.7.216

Oosthuizen MC, Zweygarth E, Collins NE, Troskie M, Penzhorn BL. Identification of a novel *Babesia sp*. from a sable antelope (*Hippotragus niger* Harris, 1838). *J Clin Microbiol*. (2008) 46:2247–51. doi: 10.1128/JCM.00167-08

Yisaschar-Mekuzas Y, Jaffe CL, Pastor J, Cardoso L, Baneth G. Identification of *Babesia* species infecting dogs using reverse line blot hybridization for six canine piroplasms, and evaluation of co-infection by other vector-borne pathogens*. Vet Parasitol*. (2013) 191:367–73. doi: 10.1016/j.vetpar.2012.09.002

Oura CAL, Bishop RP, Wampande EM, Lubega GW, Tait A. Application of a reverse line blot assay to the study of haemoparasites in cattle in Uganda. *Int J Parasitol.* (2004) 34:603–13. doi: 10.1016/j.ijpara.2003.12.012

Matjila PT, Penzhorn BL, Bekker CPJ, Nijhof AM, Jongejan F. Confirmation of occurrence of *Babesia canis vogeli* in domestic dogs in South Africa. *Vet Parasitol*. (2004) 122:119–25. doi: 10.1016/j.vetpar.2004.03.019

Bekker CP, de Vos S, Taoufik A, Sparagano OA, Jongejan F. Simultaneous detection of *Anaplasma* and *Ehrlichia* species in ruminants and detection of *Ehrlichia ruminantium* in *Amblyomma variegatum* ticks by reverse line blot hybridization. *Vet Microbiol*. (2002) 89:223–38. doi: 10.1016/S0378-1135(02)00179-7

Gubbels MJ, Hong Y, Van Der Weide M, Qi B, Nijman IJ, Guangyuan L, et al. Molecular characterisation of the *Theileria buffeli*/orientalis group*. Int J Parasitol*. (2000) 30:943–52. doi: 10.1016/S0020-7519(00)00074-6

Stoltsz H, Byaruhanga C, Troskie M, Makgabo M, Oosthuizen MC, Collins NE, et al. Improved detection of Babesia bigemina from various geographical areas in Africa using quantitative PCR and reverse line blot hybridisation. *Ticks Tick Born Dis*. (2020) 11:101415. doi: 10.1016/j.ttbdis.2020.101415

Georges K, Loria GR, Riili S, Greco A, Caracappa S, Jongejan F, et al. Detection of haemoparasites in cattle by reverse line blot hybridisation with a note on the distribution of ticks in Sicily. *Vet Parasitol*. (2001) 99:273–86. doi: 10.1016/S0304-4017(01)00488-5

Bosman AM, Venter EH, Penzhorn BL. Occurrence of *Babesia felis* and *Babesia leo* in various wild felid species and domestic cats in Southern Africa, based on reverse line blot analysis. *Vet Parasitol*. (2007) 144:33–8. doi: 10.1016/j.vetpar.2006.09.025

Schouls LM, Van De Pol I, Rijpkema SGT, Schot CS. Detection and identification of *Ehrlichia, Borrelia burgdorferi* sensu lato, and *Bartonella* species in Dutch *Ixodes ricinus* ticks. *J Clin Microbiol*. (1999) 37:2215–22. doi: 10.1128/JCM.37.7.2215-2222.1999

Schnittger L, Yin H, Qi B, Gubbels MJ, Beyer D, Niemann S. Simultaneous detection and differentiation of *Theileria* and *Babesia* parasites infecting small ruminants by reverse line blotting. *Parasitol Res*. (2004) 92:189–96. doi: 10.1007/s00436-003-0980-9
