# Supplementary material for: Porcine Babesiosis Caused by Babesia sp. Suis in a Pot-Bellied Pig in South Africa
Source: Front Vet Sci. 2021 Jan 6;7:620462. doi: 10.3389/fvets.2020.620462 (PMC7815520; doi:10.3389/fvets.2020.620462)

**Figure S1.** Representative reverse line blot results of the porcine sample. The genus- and species-specific oligonucleotides probes were applied horizontally and the PCR products, vertically. A positive- and negative control were loaded in lanes 1 and 2, respectively. The porcine sample was loaded in lanes 20 and 21. Samples loaded in lanes 3 to 19 were not relevant to this study.


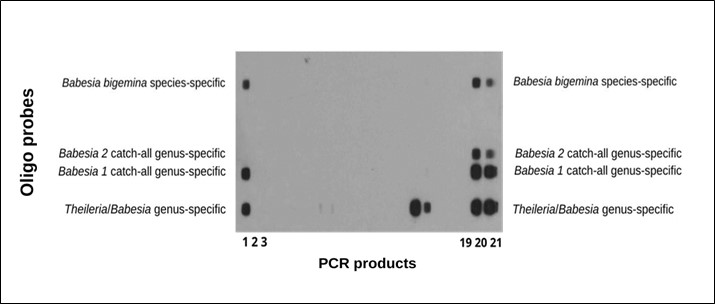

Supplement: Supplementary file 3 [file Data_Sheet_1.docx]
